# Drug-induced hypersensitivity syndrome due to phenytoin: Case report and review of the literature

**DOI:** 10.1097/MD.0000000000039715

**Published:** 2024-09-27

**Authors:** Ling Wang, Jie Zhang, Xichun Wang, Yali Xu

**Affiliations:** a Chongqing Medical University, Yuzhong District, Chongqing, China; b Department of Geriatric, Chongqing General Hospital, Chongqing University, Yuzhong District, Chongqing, China; c Chongqing Clinical Research Centre for Geriatic Diseases.

**Keywords:** antiepileptic drugs, damage of liver function, drug hypersensitivity syndrome, phenytoin sodium, systemic corticosteroids

## Abstract

**Rationale:**

Drug hypersensitivity syndrome (DIHS) is a rare but potentially fatal adverse drug reaction characterized by fever, rash, and visceral organ damage, particularly affecting the liver. Early recognition and appropriate management are crucial to prevent serious complications. However, there is limited information on the clinical presentation and management of DIHS, especially in the context of antiepileptic drugs. This case report aims to highlight the importance of recognizing subtle clinical signs and symptoms of DIHS, which can be easily overlooked, particularly in the context of antiepileptic drug use.

**Patient concerns:**

We report a case of a 15-year-old male patient who developed DIHS after being prescribed phenytoin sodium for epilepsy. The patient presented with symptoms of fever, sore throat, rash, jaundice, and liver dysfunction. Initially, the patient did not receive glucocorticoids and experienced additional reactions to cefoxitin and phosphatidylcholine, likely due to cross-reactivity.

**Diagnoses:**

The diagnosis of DIHS was made based on the patient’s clinical presentation, including fever, extensive rash, organ involvement, and hematological abnormalities. The temporal association with the use of phenytoin sodium, along with the exclusion of other causes of fever and rash, supported the diagnosis.

**Interventions:**

Upon initiation of glucocorticoid therapy with dexamethasone, the patient’s symptoms significantly improved. The rash and pruritus decreased, and laboratory values showed improvement, with a decrease in liver enzymes and normalization of white blood cell counts.

**Outcomes:**

The patient’s fever resolved within 48 hours of starting corticosteroids, and there was no evidence of ongoing inflammation as indicated by a decrease in C-reactive protein levels. Furthermore, the patient’s 30-month follow-up revealed no recurrence of rash, liver dysfunction, or organic damage, indicating the long-term effectiveness of the treatment administered.

**Lessons:**

This case highlights the importance of recognizing the subtle clinical signs and symptoms of DIHS, especially in the context of antiepileptic drug use. It underscores the potential benefits of early initiation of glucocorticoid therapy in managing DIHS. The case also serves as a reminder of the potential for drug cross-reactivity in DIHS and the need for cautious drug selection during the acute phase of the syndrome.

## 1. Introduction

Drug hypersensitivity syndrome (DIHS), also known as a drug reaction with eosinophilia and systemic symptoms, is a rare and fatal adverse drug reaction. Specific adverse reactions include fever, extensive rash and facial edema, organ involvement, and hematological abnormalities. Anticonvulsants are the cause of most cases of DIHS.^[1–3]^ Systemic glucocorticoids remain the first-line treatment for drug reaction with eosinophilia and systemic symptom (DRESS)/DIHS, while other steroid-retaining immunomodulators may be a promising treatment option, especially for refractory cases and glucocorticoid contraindications.^[1,4]^ We report a case of fever, tonsillitis, rash, epilepsy, and liver dysfunction in a 15-year-old man. The patient had been taking the antiepileptic drug phenytoin sodium for approximately 1 month prior to admission and had stopped taking antiepileptic drugs (phenytoin sodium) 1 day prior to admission. We also reviewed research on the diagnosis and treatment of DIHS and its pathogenesis, in which 2 factors (herpes virus infection and drugs) interact with the immune system to trigger the syndrome.

## 2. Case presentation

**Figure 1.**
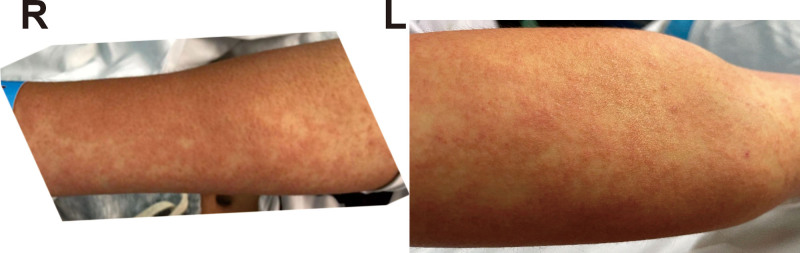
Rash and erythema on the upper limbs. R, right, L, left.

A 15-year-old boy came to our hospital with a sore throat, fever, rash of 5+ days, and jaundice of 1 day. He was taking oral nonsteroidal anti-inflammatory drugs (NSAIDS) prior to admission. He has a history of epilepsy and has been taking oral sodium phenytoin for anti-epilepsy for about 1 month. On arrival, his body temperature was 37.9°C; his body temperature raised to 39.4°C 2 hours later. Physical examination revealed many rashes throughout the body, with red papules on the limbs (Fig. 1), slightly protruding from the surface of the skin and fading with pressure. The rash on the trunk often merged into large patches or was diffusely erythematous and blanching on palpation. There were superficial lymph nodes on both sides of the neck and the submandibular region, mostly the size of peanuts, smooth, mobile and without tenderness. There was percussional pain in the area of the liver. At the time of admission, the routine blood count showed a total leukocyte count of 4.72 × 10e9/L, a monocyte count of 11.9%, C-reactive protein (CRP) of 52.82 mg/L, and a PCT of 0.64 ng/ML. Tests for respiratory pathogen profile include adenovirus antibody (IgM), influenza virus A antibody (IgM), influenza virus B antibody (IgM), *Chlamydia pneumoniae* antibody (IgM), *Mycoplasma pneumoniae* antibody (IgM), legionella antibody (IgM), parainfluenza virus antibody (IgM), respiratory syncytial virus antibody (IgM), Coxsackie virus antibody to Coxsackie virus type A (IgM), Coxsackie virus type B (IgM), Echovirus (IgM), all negative.

**Figure 2.**
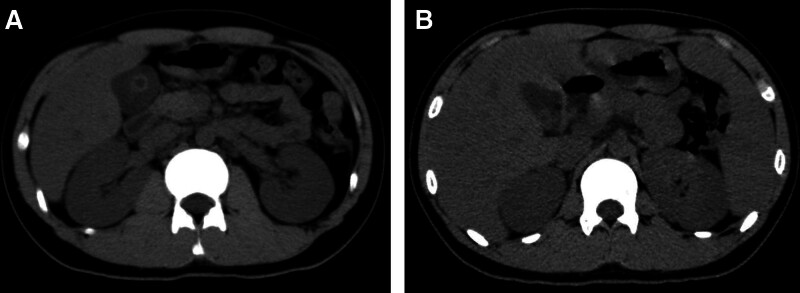
Computed tomography images on day 2 of admission showed thickening and edema of the gallbladder wall with surrounding fluid density, slightly enlarged spleen, and thickening of the perirenal fascia and lateral conal fascia of the right kidney.

Liver function showed alanine aminotransferase (ALT) 924.8 U/L, aspartate aminotransferase (AST) 713.6 U/L, total bilirubin (TBIL) 49.1 U/L, and direct bilirubin (DBIL) 47.2 U/L. The coagulation function showed PT 16.12s, INR 1.33, APTT 44.7s, FIB 238 mg/dL, and TT 19.1s. Antibodies to viral hepatitis, including hepatitis A, B, C, D, and E, were negative. Rubella virus IgG antibody 105.91 IU/mL, cytomegalovirus IgG antibody 697.76 AU/mL, herpes simplex virus type 1 IgG antibody 169.27 AU/mL; EBV antibody (serum): EBV chlamydia antigen IgG antibody test 183.00 U/mL, EBV core antigen IgG antibody test 340.00 U/mL; all IgM antibodies were negative. Ultrasound showed bilateral cervical, supraclavicular, axillary, and inguinal hypoechoic nodules consistent with lymph node enlargement; gallbladder wall is rough with cholestasis; spleen is slightly enlarged. The CT scan showed a nodule in the right upper lobe of the lung, suggesting granuloma, to be followed up. Thickening and edema of the gallbladder wall with surrounding fluid density, suggesting cholecystitis with possible accumulation of fluid in the gallbladder fossa. Slightly enlarged spleen. Thickening of the perirenal fascia and lateral conal fascia of the right kidney, suggesting inflammatory changes (Fig. 2).

**Figure 3.**
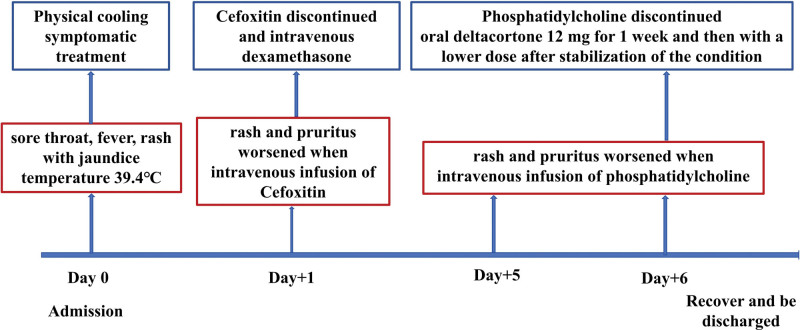
The changes in rash and pruritus and adjustments in the use of therapeutic drugs during hospitalization of the patient.

**Figure 4.**
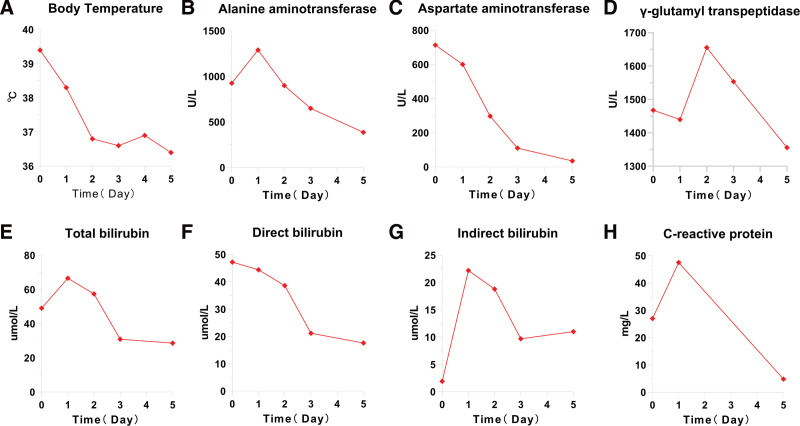
The dynamic change of body temperature (A), alanine aminotransferase (B), aspartate aminotransferase (C), γ-glutamyl transpeptidase (D), total bilirubin (E), direct bilirubin (F), indirect bilirubin (G), and C-reactive protein (H) during hospitalization of the patient.

On admission, the patient received a temporary oral solution of promethazine and ibuprofen for recurrent fever and cetirizine and desloratadine citrate disodium for cutaneous pruritus and erythra. The patient's history of purulent tonsillitis, allergic dermatitis and NSAIDS-induced liver damage was considered. The patient received cefoxitin injection for antimicrobial treatment, lamotrigine tablets for antiepileptic treatment, and sodium phenytoin was discontinued. Phosphatidylcholine injection was used for liver protection. On the 5th day after admission, there was a brief loss of consciousness, foaming in the mouth, and twitching of the limbs. Long-term recordings of EEG signals showed slow wave bursts and sharp waves. He was then given levetiracetam and the lamotrigine tablets were discontinued. The patient's rash and pruritus worsened when he received an intravenous infusion of cefoxitin injection on the second day after hospitalization. As allergic dermatitis was suspected, cefoxitin was immediately discontinued, and the patient was given 5 mg intravenous dexamethasone. On the 5th and 6th day of hospitalization, the patient experienced increased pruritus and rash with intravenous phosphatidylcholine, which was discontinued. The patient was then treated with oral deltacortone 12 mg for 1 week and then with a lower dose after stabilization of the condition. Figure 3 showed the changes in rash and pruritus and adjustments in the use of therapeutic drugs during the patient's hospitalization. Liver function at discharge: ALT: 386.9 U/L, AST: 35.2 U/L, total bilirubin: 28.6 umol/L, directdirect bilirubin: 17.6 umol/L. The dynamic change of body temperature, liver function and CRP were shown in Figure 4. During the 30-month follow-up, the patient did not experience rash, liver dysfunction or visual organic damage.

## 3. Outcomes

Upon initiation of oral dexamethasone and subsequent systemic corticosteroid therapy, the patient's symptoms improved significantly. The rash and pruritus decreased. Laboratory values also showed improvement, with a decrease in liver enzymes and normalization of white blood cell counts. During treatment, the patient ALT and AST levels dropped from initial values of 924.8 U/L and 713.6 U/L, respectively, to 386.9 U/L and 35.2 U/L at the time of discharge. Total bilirubin levels also decreased from 49.1 μmol/L to 28.6 μmol/L. The patient's fever resolved within 48 hours of starting corticosteroids, and there was no evidence of ongoing inflammation as indicated by a decrease in CRP levels. Furthermore, the patient's 30-month follow-up revealed no recurrence of rash, liver dysfunction, or organic damage, indicating the long-term effectiveness of the treatment administered.

## 4. Discussion

We experienced a case with typical clinical features of DIHS and, to our knowledge, anticonvulsants are the most common noncompliant drug. Among these, sodium phenytoin is the most common drug.^[5,6]^ The patient has a clear history of use of antiepileptic, antipyretic, and analgesics prior to illness. Initially, DIHS was not recognized and systemic corticosteroids were not administered in a timely manner; hepatoprotective drugs and cephalosporins were administered in the preceding days. However, during the use of cephalosporins and hepatoprotective drugs, the patient experienced repeated exacerbations of rash and pruritus. After temporary use of dexamethasone, symptoms improved, but when cephalosporins and hepatoprotective drugs were resumed, symptoms worsened again. Withdrawal of hepatoprotective agents and cephalosporins and systemic corticosteroids were administered, and symptoms improved and then disappeared.

The pathogenesis of DIHS is currently unclear and is associated with the reactivation of human herpesvirus 6 (HHV-6),^[1,7]^ which is a potentially fatal multiorgan hypersensitivity reaction associated with the reactivation HHV-6. We have not tested the HHV-6. A positive correlation between the expression of the HHV-6 receptor and the DIHS/DRESS severity (DDS) score suggested a possible role for HHV-6 and its receptor in the mechanism underlying the progression and pathophysiology of DIHS.^[8]^

Fever is the most common symptom, followed by rash,^[9]^ Liver involvement is the most common visceral manifestation of DIHS/DRESS and can lead to fulminant liver failure and death.^[10]^ Most patients have an incubation period of more than 14 days, and the common clinical features include elevated eosinophil count/percentage, fever, rash, liver damage, and enlarged lymph nodes.^[11]^ DIHS shares similarities with other systemic inflammatory syndromes (notably systemic juvenile idiopathic arthritis, macrophage activation syndrome, and secondary hemophagocytic lymphohistiocytosis).^[12]^ These conditions share clinical features such as fever, rash, lymphadenopathy, internal organ involvement, and cytopenia (or hyperleukocytosis in the case of systemic juvenile idiopathic arthritis).^[12]^

This case is consistent with the triad of fever, rash, and internal organ damage (especially liver) that often occurs 7 to 28 days or longer after first taking the drug.^[13]^ The patient had been taking sodium phenytoin for about a month. Typically, up to 70% of patients with DIHS present with various abnormalities of liver function, ranging from mild elevation of ALT levels to fulminant liver failure, but most patients do not typically present with jaundice. This case presents with rare clinical features of DIHS, with ALT, AST, and ALP more than 3 times normal, accompanied by deep jaundice, suggesting severe liver injury. Systemic corticosteroids are recognized as the standard of care for improving clinical symptoms in the acute phase of DIHS.^[14,15]^ Developing treatments that can reduce the risk of fatal complications and subsequent autoimmune disease in patients seems to be a reasonable approach.^[16]^

During the acute phase of DIHS, there is a high risk of cross-reactivity of the drug. DIHS usually shows unexplained cross-reactivity to several drugs (including those used after onset). Patients experience systemic rash after taking ibuprofen or other drugs before administration. The use of cefoxitin and phosphatidylcholine has worsened symptoms, which may be caused by drug cross-reactivity.

## 5. Limitations

While our case study provides valuable insights into the management of DIHS, this case report has several limitations. Firstly, it is a single-case study, which limits the generalizability of the findings. Secondly, we did not test for HHV-6 reactivation, a potential pathogenic factor in DIHS, which could have provided further understanding of the patient condition. Finally, the cross-reactivity observed with other drugs such as cefoxitin and phosphatidylcholine was not systematically studied, which could have implications for the broader understanding of drug interactions in DIHS.

## 6. Conclusion

We report a case with typical clinical features of DIHS. ALT, AST, and ALP are more than 3 times normal, accompanied by deep jaundice. If patients receiving anticonvulsant treatment have these symptoms, DIHS should be suspected. Systemic corticosteroids are important. This case may provide an empirical reference for the early recognition and management of antiepileptic drug-induced DIHS in clinical practice, avoiding unnecessary interventions and prolonged hospitalization.

## Acknowledgments

The authors appreciate the patient and the guardians of the patient.

## Author contributions

**Data curation:** Jie Zhang, Xichun Wang, YALIYALI Yali Xu.

**Formal analysis:** Jie Zhang, Xichun Wang.

**Funding acquisition:** YALIYALYali Xu.

**Resources:** Jie Zhang, Xichun Wang.

**Supervision:** Jie Zhang, Yali Xu.

**Writing: original draft:** Ling Wang.

**Writing—review & editing:** Ling Wang, Yali Xu.
